# Photo-Thermal Conversion and Raman Sensing Properties of Three-Dimensional Gold Nanostructure

**DOI:** 10.3390/molecules29184287

**Published:** 2024-09-10

**Authors:** Feng Shan, Jingyi Huang, Yanyan Zhu, Guohao Wei

**Affiliations:** 1 Department of Mathematics and Physics, Luoyang Institute of Science and Technology, Luoyang 471023, China; 2 Henan Key Laboratory of Green Building Materials Manufacturing and Intelligent Equipment, Luoyang Institute of Science and Technology, Luoyang 471023, China; 3 School of Environmental Engineering and Chemistry, Luoyang Institute of Science and Technology, Luoyang 471023, China; m18317412398@163.com (J.H.); 15518136314@163.com (Y.Z.); 18137761712@163.com (G.W.)

**Keywords:** nanoparticles, photo-thermal, electrophoretic deposition, local electric field, SERS

## Abstract

Three-dimensional plasma nanostructures with high light–thermal conversion efficiency show the prospect of industrialization in various fields and have become a research hotspot in areas of light–heat utilization, solar energy capture, and so on. In this paper, a simple chemical synthesis method is proposed to prepare gold nanoparticles, and the electrophoretic deposition method is used to assemble large-area three-dimensional gold nanostructures (3D-GNSs). The light–thermal water evaporation monitoring and surface-enhanced Raman scattering (SERS) measurements of 3D-GNSs were performed via theoretical simulation and experiments. We reveal the physical processes of local electric field optical enhancement and the light–thermal conversion of 3D-GNSs. The results show that with the help of the efficient optical trapping and super-hydrophilic surface properties of 3D-GNSs, they have a significant effect in accelerating water evaporation, which was increased by nearly eight times. At the same time, the three-dimensional SERS substrates based on gold nanosphere particles (GNSPs) and gold nanostar particles (GNSTs) had limited sensitivities of 10^−10^ M and 10^−12^ M to R6G molecules, respectively. Therefore, 3D-GNSs show strong competitiveness in the fields of solar-energy-induced water purification and the Raman trace detection of organic molecules.

## 1. Introduction

In recent years, three-dimensional plasma nanostructures with optical trapping and photo-thermal conversion properties have become a field of great interest. They have shown bright application prospects in many fields, such as photothermal utilization [1,2], surface-enhanced Raman scattering (SERS) [3,4,5], solar energy enhancement [6,7,8], light treatment [9,10], ultraviolet protection [11], stealth [12,13], and photocatalysis [14,15]. The above applications are mainly related to the local surface plasmon resonance (LSPR) properties of metal nanoparticles. Three-dimensional thin-film structures composed of metal nanoparticles with this property show multiple physical effects [16,17,18]. At the beginning, the LSPR excitation of the three-dimensional plasma nanostructures enhances the photon-energy absorption, and the yield of “hot electrons” produced in the excited state can be significantly increased. Both radiative and non-radiative decay will occur in the subsequent decay process of hot electrons. Among them, radiation attenuation will lead to the re-emission of light, the intensity of which has been significantly increased. These re-emitted lights are either scattered into free space or captured into the near-field volume, with an intensity enhancement of several orders of magnitude [18,19,20,21]. It is thanks to this electromagnetic enhancement property that the three-dimensional plasma nanostructures have a great advantage in the field of SERS sensing [22]. The phenomenon of light absorption caused by non-radiation attenuation is also a manifold process [18,19]. Photon energy can be converted into electron energy by electron–electron scattering, and it can also be converted into thermal energy by vibrational resonance effects [19,23]. Therefore, the three-dimensional plasma nanostructure can effectively convert visible light and near-infrared light into electron energy or thermal energy, which has attracted wide attention in the field of solar energy utilization [6,7,8]. Thus, it is in line with the development of renewable and environment-friendly technology in the future. These multiple physical effects are highly dependent on the size and morphological characteristics of plasma nanostructures [24,25]. At present, the research is mainly focused on how to prepare three-dimensional plasma nanostructures with excellent optical enhancement properties via the morphological design method.

The three-dimensional metal-nanoparticle-based thin films prepared by means of nanolithography have important advantages in terms of their structural uniformity [26,27]. However, considering the low cost and time-saving properties, the three-dimensional metal nanostructures prepared via chemical methods have broader industrial prospects. If we want to prepare three-dimensional metal nanostructures with excellent SERS sensing properties, it is very important to ensure the strong electromagnetic enhancement of the surfaces of metal nanoparticles. The electromagnetic enhancement of the surfaces of metal nanoparticles strongly depends on their morphological characteristics. Metal nanoparticles with tip structures will give rise to a hot spot effect on their surface, resulting in greater electromagnetic enhancement. At present, with the rapid development of chemical synthesis technology, various forms of metal nanoparticles have been successfully synthesized, such as nanospheres, nanocubes, nanorods, nanotriangles, nanoflakes, and nanoflowers [28,29,30,31,32,33]. Tian’s group [34] studied the effects of the morphology of gold nanoparticles on the enhancement properties of SERS. They chose spherical, triangular-plate, and star-shaped metal nanoparticles with three different morphologies for Raman testing. When the dye R6G was selected as the analyte, the Raman spectrum intensity of gold nanostar SERS substrate was the highest, followed by the gold nano-triangle plate and gold nanospheres. Qian’s team [35] studied the SERS substrates of gold nanoparticles with different branching lengths. Under the same experimental conditions, the results show that the surface enhancement factor of the SERS substrate is proportional to the surface branching length of gold nanoparticles. However, compared with the local field enhancement characteristics formed on the surfaces of individual metal nanoparticles, the hot spots formed by the coupling effects between particles have stronger electromagnetic field enhancement effects [36,37,38]. On the other hand, the hot spots formed by the coupling effect between multi-tip metal nanoparticles have a higher intensity. At the same time, three-dimensional structures based on multi-tip metal nanoparticles can provide a larger number of hot spots. The application of this structure in photothermal conversion and SERS sensing is of great significance.

Here, we propose a three-dimensional nanostructure based on gold nanosphere particles (GNSPs) and multi-tip gold nanostar particles (GNSTs). GNSTs with a yield of almost 100% were prepared by means of multi-step chemical synthesis. On this basis, we prepared a three-dimensional gold nanostructure (3D-GNS) with super-hydrophilic properties on an ITO glass substrate via the electrophoretic deposition technique. In the process of deposition, the voltage and working time were optimized, and a 3D-GNS with good uniformity was obtained. Theoretical analysis shows that there is a coupling effect between particles in the 3D-GNS, with strong local enhancement in the near field. Due to the unique optical properties of this structure, it has extremely important application value in photo-thermal conversion and SERS enhancement.

## 2. Results and Discussion

### 2.1. Characterization of Gold Seed Nanoparticles

**Figure 1:**
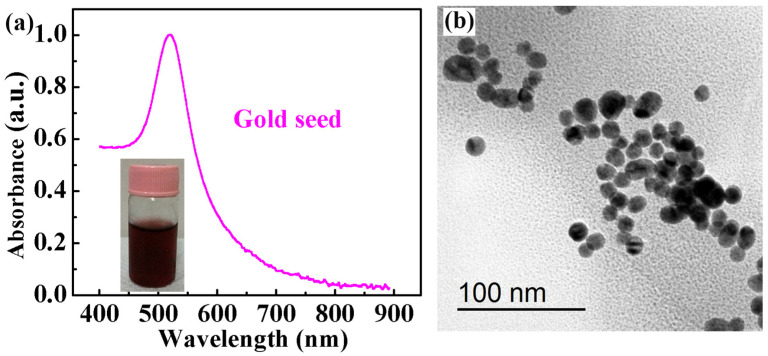
(**a**) The absorption spectrum of the gold seed solution (inset: gold seed solution). (**b**) The TEM image of gold seed nanoparticles.

The synthesis of gold nanoparticles includes two processes: the synthesis of gold seeds and the growth of gold nanoparticles. Therefore, excellent gold seed properties are very important for the preparation of gold nanoparticles. HAuCl_4_·3H_2_O in a boiling state reacts with trisodium citrate. Thus, the gold seed nanoparticles are obtained. The gold seed solution was wine red, as shown in the inset of Figure 1a. Firstly, the optical characteristics of the gold seed solution were tested. The absorption spectrum of the gold seed solution measured using the optical fiber spectrometer is shown in Figure 1a. We can see from the absorption curve that the wavelengths were very flat before 450 nm, and then they rose rapidly. The peak of the absorption spectrum appeared at the wavelength of 520 nm. Then, the absorption spectral curve dropped rapidly. The curve became relatively flat after the wavelength reached 800 nm. We used TEM to further test its morphology image, as shown in Figure 1b. It can be clearly seen from the image that most of the gold seeds had a spherical structure. The sizes of the gold seed nanoparticles were mostly between 10 and 20 nm. Although there were a small number of nanoparticles with larger sizes, the proportions were very small. This shows that the gold seeds we synthesized were of good quality. This will provide an important guarantee for the synthesis of gold nanoparticles.

### 2.2. Characterization of GNSPs and GNSTs

**Figure 2:**
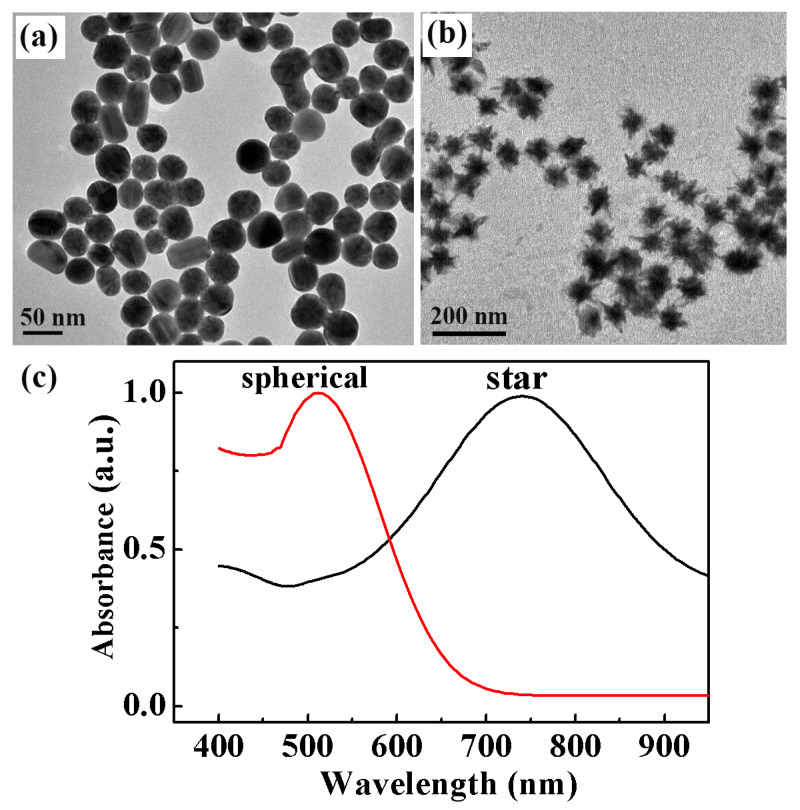
SEM images of (**a**) GNSPs and (**b**) GNSTs. (**c**) Absorption spectra of GNSP and GNST solutions.

From the schematic illustration of gold nanoparticle synthesis in Figure 8, it can be seen that AgCl plays an important role in the growth of tip branches on the surface of gold nanoparticles. Once AgCl precipitates are adsorbed onto the surface of gold seed nanoparticles, the adsorption position will no longer grow. On the contrary, the sites without AgCl adsorption will continue to grow, resulting in multi-tip dendritic GNSTs. If we did not add AgNO_3_ when synthesizing GNSTs, the surfaces of gold seed nanoparticles grew uniformly, and finally the GNSPs were obtained. The TEM images of the GNSPs and GNSTs are shown in Figure 2a,b, respectively. It can be seen from the figure that both achieved good nanoparticle dispersion. In Figure 2a, we see that the morphology of the nanoparticles was mainly spherical, while a few non-spherical particles, such as nanorods and nanoellipsoids, appeared at the same time. A tip structure developed on the surface of the nanoparticles, as shown in Figure 2b; here, the yield of gold nanoparticles was close to 100%, and the size uniformity was excellent. Gold nanoparticles with different morphologies must have different optical properties. Subsequently, we measured the absorption spectra of two kinds of nanoparticle solutions with the help of a spectrometer, as shown in Figure 2c. It can be clearly seen from the picture that the absorption spectrum of GNSPs was similar to that of gold seed particles, and the resonance peak was near 520 nm. On the other hand, the resonance peak of the absorption spectrum of GNSTs had an obvious red shift, reaching about 750 nm. Another obvious difference was the FWHM (full width at half maxima) of the absorption spectra. Compared with that of GNSPs, the absorption spectrum of GNST had a larger FWHM. The resonance peak of metal nanoparticles can be controlled by changing their morphology and size. These control methods provide important technical support for the application of metal nanoparticles in the fields of fluorescence enhancement, SERS, optical devices, and other fields.

### 2.3. Theoretical Properties of GNSPs and GNSTs

As shown in Figure 2, we experimentally prepared GNSPs and GNSTs. Next, wefurther theoretically analyzed the optical properties of the GNSPs and GNSTs. In the process of building the model, the radius of the GNSPs was 30 nm, the radius of the core of the GNSTs was 30 nm, and the tip length of the GNSTs was 20 nm. The refractive index parameters for the GNSPs and GNSTs were taken from the literature [39]. The dielectric constant around the gold nanoparticles was consistent with the parameters of water, and the refractive index was 1.33. During the simulation, performed with the help of the finite element method, the grid at the gap position was set to 0.2 nm, and the free space was set to 2 nm. This not only ensured the accuracy of the calculation, but also saved the time spent in the calculation. A perfectly matching layer was placed around the model to completely absorb the electromagnetic waves scattered to the edges, thus ensuring the reliability of the calculation’s results. In the inner space of the metal nanoparticle film, the distance between nanoparticles was in the order of nanometers, and a coupling effect occurred between them. As such, we not only simulated the local electric field’s distribution on the surface of a single gold nanoparticle but also calculated the electric field distribution at the point where the coupling effect occurred between two nanoparticles.

**Figure 3:**
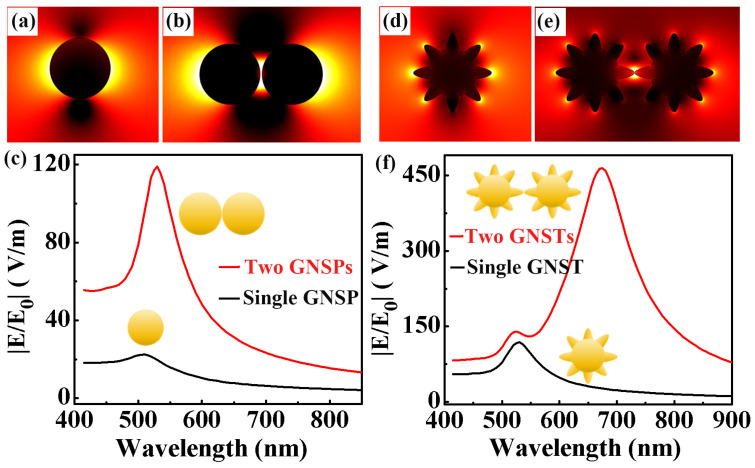
Electric field distributions on the surface of (**a**) a single GNSP and (**b**) two GNSPs. (**c**) Electric field resonance spectra on the surface of a single GNSP and two GNSPs. Electric field distributions on the surface of (**d**) a single GNST and (**e**) two GNSTs. (**f**) Local electric field resonance spectra on the surface of a single GNST and two GNSTs.

Figure 3a shows an image of the local electric field distribution image on the surface of GNSPs. It can be seen from the figure that the electric field was mainly localized on the surface of the nanoparticles and was symmetrically distributed on both sides of the GNSPs. Rapid decay occurred far from the nanoparticle’s surface. Figure 3b shows an image of the local electric field distribution image on the surfaces of two GNSPs. It can be seen from the figure that the local electric field was mainly distributed in the gap between the GNSPs. Although there was also an electric field distribution on the sides of the two GNSPs, its intensity was much smaller than the electric field intensity in the gap region. As shown in Figure 3c, we calculated the local electric field resonance spectra of both a single and two GNSPs. The maximum electric field intensity of a single GNSP is about 30 V/m. The wavelength of the resonance peak is located around 520 nm, which is consistent with the experimental data shown in Figure 2c. The local electric field resonance spectrum formed by two GNSPs has a larger intensity value. It can be seen from the red line in Figure 3c that it was about 120 V/m. This was mainly due to the coupling effect between the two GNSPs. At the same time, we also found that the resonant peak wavelengths of the two GNSPs also underwent a red shift.

Under the same simulation conditions, we also calculated the local electric field enhancement characteristics of both a single and two GNSTs. Figure 3d,e show their local electric field distributions. Like that for GNSPs, the electric field was localized on the surface of the GNST and was symmetrically distributed on both sides of the particle. For a single GNST, hot spots formed at the tip. For two GNSTs, the electric field was mainly localized in the gap region between the GNSTs. It can be seen in Figure 3f that two resonant peaks appeared in the local electric field resonance spectra of two GNSTs, where the intensity of the main resonant peak was much greater than the local electric field intensity of a single GNST. The resonant peak wavelength showed a significant redshift of about 180 nm. It can be seen from the above simulation results that with the excitation of incident light, the electric field of gold nanoparticles was mainly localized onto the particles’ surface. A strong coupling effect occurred between two gold nanoparticles, and the local electric field intensity obtained in the gap region between the two nanoparticles was much greater than the electric field intensity on the surface of a single gold nanoparticle. Thus, we successfully experimentally prepared high-yield, monodisperse GNSPs and GNSTs. To study the coupling effect between nanoparticles and obtain a gold nanoparticle structure with a higher local electric field, we experimentally prepared 3D-GNSs.

### 2.4. Characterization of 3D-GNSs

**Figure 4:**
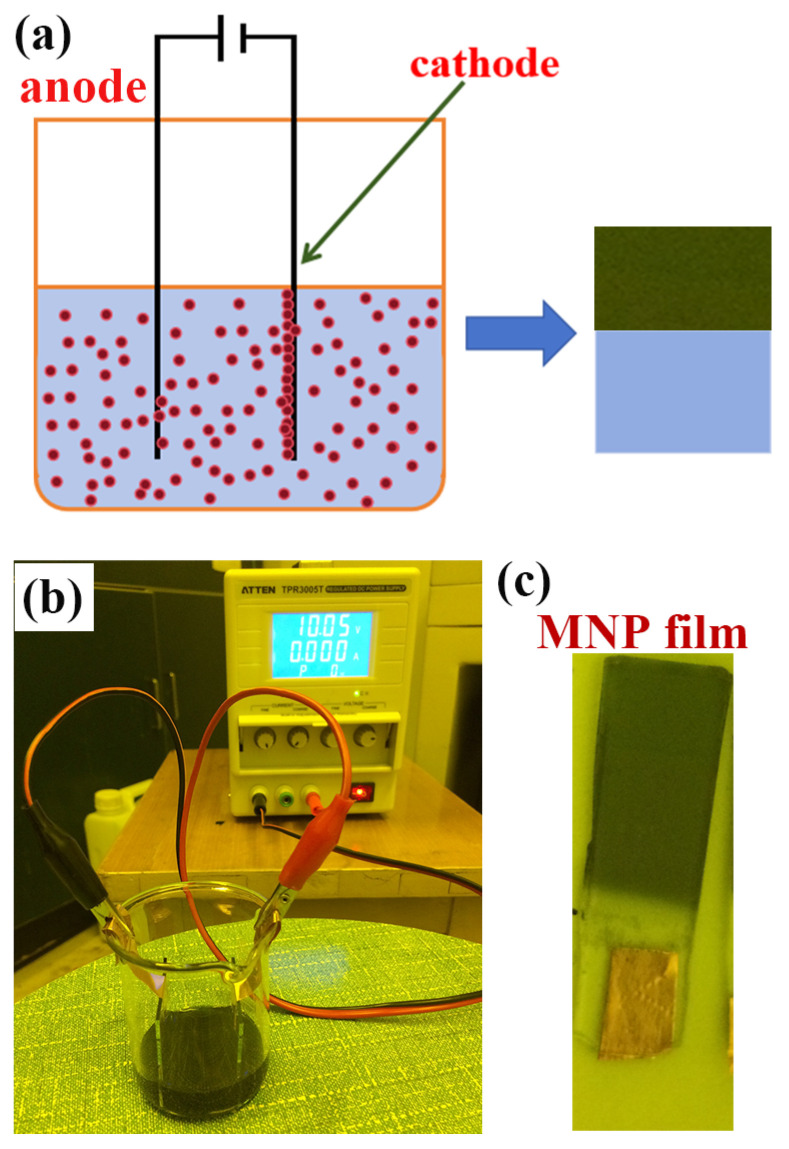
(**a**) Schematic illustration of the electrophoretic deposition method. (**b**) Experimental equipment used for assembling 3D-GNSs via electrophoretic deposition. (**c**) Physical picture of 3D-GNSs assembled via electrophoretic deposition.

The uniformity of a 3D-GNS has an important impact on the detection of Raman spectral signal stability. We used the electrophoretic deposition method to prepare 3D-GNSs; Figure 4a shows a schematic image of the electrophoretic deposition method. We chose DC as the power supply device for electrophoretic deposition. Selecting the appropriate voltage for electrophoretic deposition experiments can not only help ensure that we obtain a uniform 3D-GNS but can also keep the morphology of gold nanoparticles unchanged. Figure 4b shows the experimental device used for assembling gold nanoparticle films via electrophoretic deposition and pictures of the prepared film samples. The voltage selected during the experiment was 10 V, and the substrate was ITO glass. We used conductive copper tape to connect to the ITO glass and fix the copper tape to the edge of the beaker mouth. We then tightly clamped the copper tape with an alligator clip. Red represents the anode and black represents the cathode. ITO glass was vertically placed into a well-dispersed gold nanoparticle solution, and after 30 min of electrophoretic deposition, a gold nanoparticle thin-film structure was deposited on the surface of the ITO glass at the cathode. Figure 4c shows the structure of the thin film deposited with gold nanoparticles. It can be seen from the figure that the gold nanoparticles had excellent uniformity, indicating that the voltage we used was appropriate. This will greatly benefit our subsequent Raman signal stability testing of 3D-GNS SERS substrates. To determine whether the morphology of gold nanoparticles has changed, it is still necessary to use SEM for testing and characterization.

**Figure 5:**
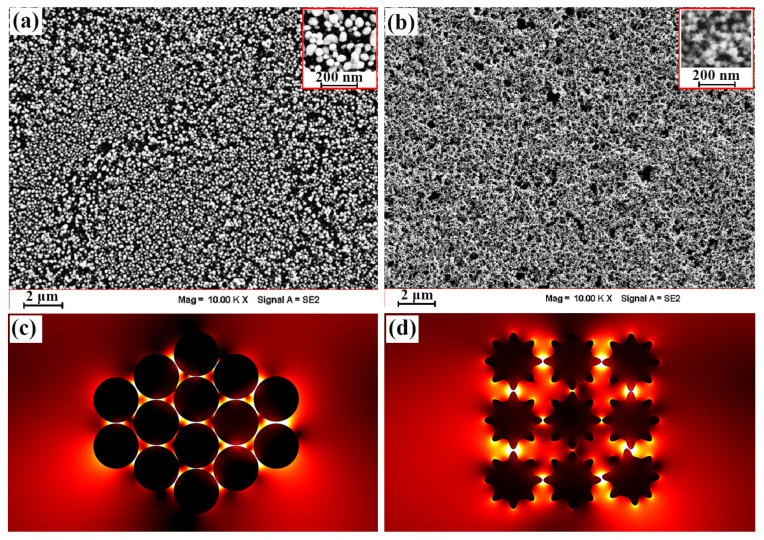
SEM images of (**a**) GNSP films and (**b**) GNST films. Images of the coupling effect within the electric field distribution for (**c**) GNSPs and (**d**) GNSTs. The insets in (**a**,**b**) are higher-magnification SEM images.

We successfully prepared a GNSP 3D-GNS and a GNST 3D-GNS using electrophoretic deposition. However, during the deposition process, it was very important to preserve the morphology of metal nanoparticles and the uniformity of the film. Next, we tested the surface characteristics of the film with SEM, and the results are shown in Figure 5a,b. Overall, both gold nanoparticle films maintained excellent uniformity, which shows that the deposition voltage and time we selected were appropriate. In addition, it can be seen from the figure that their morphology characteristics were also very well preserved. Before and after the film’s deposition, the morphology of the particles did not change. As shown in Figure 5a, GNSPs can be clearly seen in the film. The morphology of the GNSTs can also be distinguished in Figure 5b. However, the surface of the GNSTs featured a tip structure, and when they were close to each other, they were more tightly embedded, making the film look more compact. In Figure 3, we have theoretically demonstrated that the coupling effect between gold nanoparticles greatly increases the intensity of the local electric field on their surfaces. Thus, we further simulated the coupling effect between the three-dimensional gold nanoparticles in the layered structure, as shown in Figure 5c,d. It can be seen from the figure that many “hot spots” formed in the gaps between nanoparticles that were close to each other. The electric field strength of these hot spot structures was much greater than that in other areas, and it played an important role in the fields of photo-thermal conversion and SERS detection. More importantly, each particle in the 3D-GNS will form multiple hot spot structures with surrounding nanoparticles, and so the entire 3D-GNS will provide a sufficient number of hot spot structures. This feature will provide great benefits in subsequent photo-thermal conversion and SERS applications based on gold nanoparticle films.

### 2.5. Light-Heat Conversion Characteristics of the 3D-GNSs

**Figure 6:**
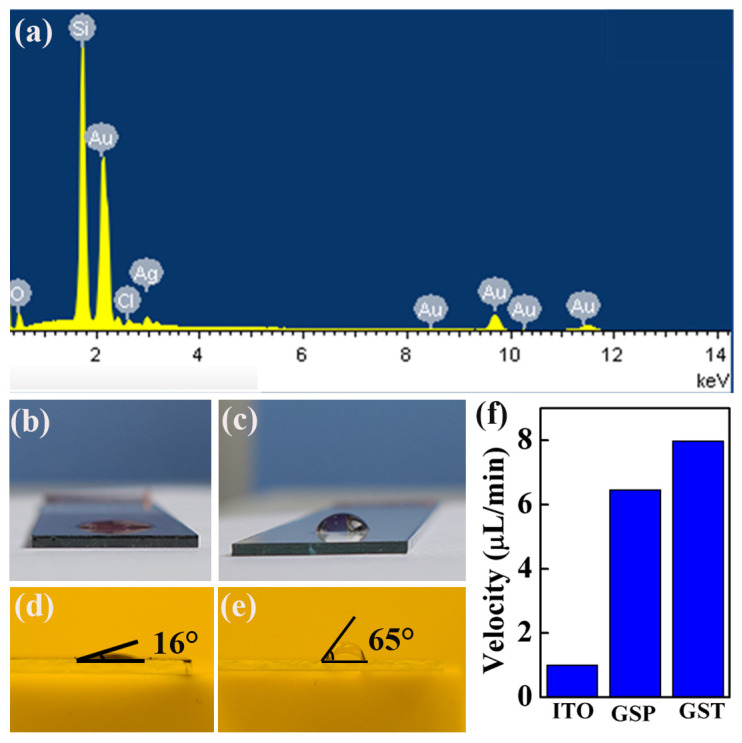
(**a**) EDS spectrum of 3D-GNS. (**b**) Shape of water droplets on the surface of 3D-GNS. (**c**) Shape of water droplets on the surface of ITO glass. (**d**) Water drop contact angle on the 3D-GNS surface. (**e**) Water drop contact angle on the ITO glass surface. (**f**) Water droplet evaporation rate on the ITO glass and 3D-GNS surfaces.

Since the preparation of gold nanoparticles involves two processes, seed synthesis and nanoparticle growth, a variety of chemical reagents are used during this process. The more critical fact is that AgCl precipitates are adsorbed onto the surface of GNSTs during the growth process. Therefore, we used EDS (energy dispersive spectrometer) technology to analyze the elemental composition of the 3D-GNS, as shown in Figure 6a. It can be seen from the figure that the elements with the highest abundance were silicon and gold; silicon comes from the silicon substrate used for testing and cannot be counted. Therefore, the EDS spectroscopy results show that the main component of the 3D-GNS is gold. As for the Cl, Ag, and O elements therein, they come from related reagents used in the redox reaction process. It can be seen from the EDS spectrum that their proportions are extremely small.

Under the excitation of incident light, plasmon excitons generated by the 3D-GNS can be converted into thermal energy. Therefore, solar light–heat utilization based on the 3D-GNS can be applied in various fields, and this technology has the advantages of green environmental protection. Next, we used the light–heat conversion characteristics of the 3D-GNS to quantitatively study its ability to accelerate water evaporation. The hope is to bring about new applications in the field of seawater desalination. In order to be consistent with actual applications, we chose a standard AM1.5 spectrum irradiation. Studies have shown that under the condition of a light source power of 5 kW/m^2^, the surface temperature of a 3D-GNS can increase from room temperature to above 75 °C after 5 min of irradiation, and then tends to stabilize [13]. Under the same light source irradiation conditions, the temperature of the exposed ITO glass surface did not increase much. As shown in Figure 6b, we can see that the 3D-GNS has super-hydrophilic characteristics. The exposed ITO glass has hydrophobic properties, as shown in Figure 6c. We then measured their contact angles, which were 16° and 65° for the 3D-GNS based on GNSTs and bare ITO glass, respectively. In order to test the water-volatilization ability of the 3D-GNS, we conducted a comparative experiment. A 550 W halogen lamp was used to simulate sunlight, set 10 cm away from the 3D-GNS. We took 2000 μL of water and dropped it continuously onto the 3D-GNSs based on GNSPs and GNSTs, as well as the surface of the exposed ITO glass. The surface had to be kept moist at all times during the water evaporation process. We defined the water evaporation rate as 2000 μL of water/total time of the evaporation process, and the results are shown in Figure 6f. From the results, we can see that the water evaporation rate of the 3D-GNS based on GNSTs was 8 μL/min; the water evaporation rate of the 3D-GNS based on GNSPs came second; and the water evaporation rate of bare ITO glass was the lowest, at only about 1 μL/min. Obviously, the super-hydrophilic nature of the 3D-GNS can enhance the light–heat conversion efficiency and further increase the rate of water evaporation. This offers an idea for designing purification equipment with new water evaporation methods.

### 2.6. SERS Characteristics of the 3D-GNS

Another important application of the local electric field enhancement characteristics of the 3D-GNS is SERS. The Raman spectral signal of R6G molecules adsorbed on the surface of the 3D-GNS is greatly enhanced by the local electric field. We prepared SERS samples by immersing the SERS substrate in R6G solution, such that the R6G molecules could be uniformly attached to the surface of the SERS substrate. First, a certain concentration of R6G ethanol solution was prepared, and the concentration of R6G was adjusted according to the experimental requirements. We stirred this well so as to ensure that the R6G was completely dissolved. Secondly, the prepared SERS substrate was completely immersed in the R6G solution for 30 min. Then, R6G molecules were adsorbed on the surface of the SERS substrate. Immediately afterwards, the SERS substrate on which the R6G molecules were adsorbed was naturally dried. Finally, the dried SERS substrate was placed under a Raman spectrometer for testing, thereby obtaining enhanced Raman signals.

**Figure 7:**
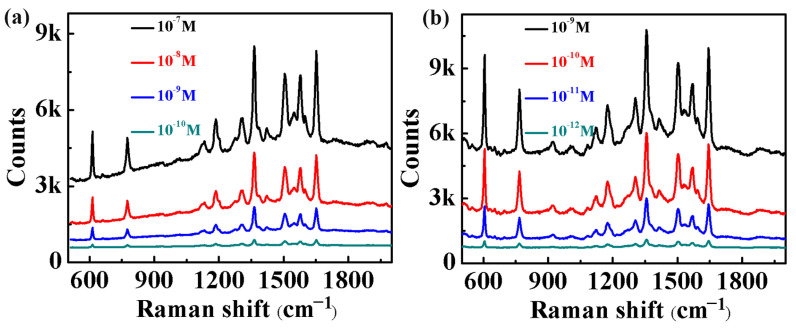
(**a**) Raman spectra of R6G molecules on 3D-GNS SERS substrate based on GNSPs. (**b**) Raman spectra of R6G molecules on 3D-GNS SERS substrate based on GNSTs.

In Figure 3, it is shown that the local electric field intensity in the gap region between gold nanoparticles is much greater than the intensity on the surface of a single gold nanoparticle. The 3D-GNSs we prepared were composed of a large number of gold nanoparticles close to each other, and a large number of gap structures were formed between the nanoparticles. Under the excitation of incident light, the electric field in the gap region was greatly enhanced due to the hot spot effect. Therefore, the 3D-GNSs are very suitable for use as a SERS substrate for the Raman signal detection of organic molecules. As shown in Figure 7a, we measured the Raman signal of R6G molecules based on a 3D-GNS SERS substrate of GNSPs. It can be seen from the figure that the intensity of the Raman spectral signal of R6G molecules decreased with the decrease in the molecular concentration, and the limit concentration of R6G molecules was 10^−10^ M. Under the same test conditions, we also tested the Raman spectral properties of 3D-GNS SERS substrates based on GNSTs. We found the same trend as shown in the figure, that is, the higher the concentration of R6G molecules, the stronger the Raman spectrum signal obtained. However, the lowest test concentration for testing the Raman signal of R6G molecules based on the 3D-GNS structure SERS substrate of GNSTs was 10^−12^ M. The results show that the 3D-GNSs composed of GNSTs had stronger local electromagnetic field enhancement characteristics than those composed of GNSPs, which is consistent with the simulation results shown in Figure 3.

## 3. Experimental

### 3.1. Chemicals

Ascorbic acid (AA, >99.5%), silver nitrate (AgNO_3_, 99.9%), trisodium citrate (≥99.9%), and gold chloride trihydrate (HAuCl_4_·3H_2_O, >99.9%) were purchased from Sigma-Aldrich, Cleveland, OH, USA. Hydrochloric acid (HCl, >98.0%) was purchased from Sinopharm Chemical Reagent Co., Ltd., Shanghai, China. Ultrapure water from Milli-Q equipment (Millipore, Burlington, MA, USA; resistivity > 18.2·MΩ cm) was used throughout the experiments. All materials were used without further purification.

### 3.2. Characteristics

The morphological characteristics of gold seed nanoparticles, GNSPs, and GNSTs were measured via TEM (Fei Tecnai T20, Hillsboro, OR, USA). The three-dimensional structural characteristics and EDS of gold nanoparticles were tested via SEM (Zeiss Ultra Plus, Oberkochen, Germany). The absorption spectrum of the gold nanoparticle solution was measured using an optical fiber spectrometer (NOVA, Ideaoptics Technology Ltd., Shanghai, China). The SERS signals of gold-nanoparticle-based three-dimensional thin films were measured using a Raman spectrometer (LCRMS, LabRAM HR UV-Visible, Paris, France). The wavelength and power of the laser were 532 nm and 100 mW, respectively. The acquisition time was 10 s.

### 3.3. Methods

**Figure 8:**
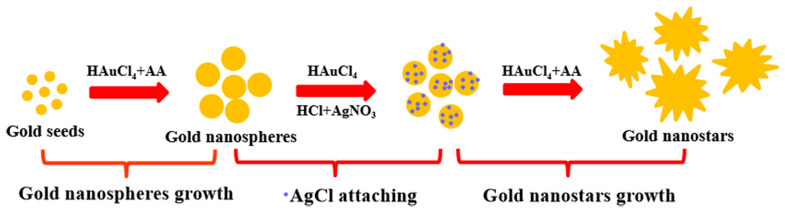
Schematic illustration of the synthesis of GNSTs.

We used a two-step method to synthesize GNSPs and GNSTs, as shown in Figure 8. The first step was to synthesize the gold seed solution. The second step involved the growth of gold seed nanoparticles to prepare GNSP and GNST. The specific process is as follows.

#### 3.3.1. Synthesis of Gold Seeds

First, 15 mL of trisodium citrate solution with a mass fraction of 1% and 100 mL of HAuCl_4_·3H_2_O solution with a concentration of 1 mM were prepared. Then, the HAuCl_4_·3H_2_O solution was heated to boiling. Next, while stirring the HAuCl_4_·3H_2_O, we quickly added trisodium citrate solution to it. We then continued stirring for 15 min to allow the HAuCl_4_·3H_2_O and trisodium citrate to fully react, and the solution ultimately turned wine red. After cooling, this could be used as the gold seeds solution for the preparation of GNSPs and GNSTs.

#### 3.3.2. Synthesis of GNSP

A HCl solution with a 100 μL concentration of 1 M was added to the HAuCl_4_·3H_2_O solution with a 100 mL concentration of 0.25 mM. Then, the mixed solution was stirred at 700 rpm. Next, 1 mL of gold seed solution was added to the HAuCl_4_·3H_2_O mixed solution, and stirring was continued for 5 min. Finally, 500 μL of 100 mM AA solution was added to the mixture. After 30 s of reaction, GNSPs were synthesized.

#### 3.3.3. Synthesis of GNST

A HCl solution with a 100 μL concentration of 1 M was added to the HAuCl_4_·3H_2_O solution with a 100 mL concentration of 0.25 mM. Then, the mixed solution was stirred at 700 rpm. Next, 1 mL of gold seed solution was added to the HAuCl_4_·3H_2_O mixed solution, and stirring was continued for 5 min. Finally, 500 μL of 100 mM AA solution and 1 mL of 0.01 M AgNO_3_ solution were added to the mixture simultaneously. After 30 s of reaction, GNSTs were synthesized.

The 3D-GNS was prepared by means of electrophoretic deposition. The successfully prepared gold nanoparticles were centrifuged at a speed of 4500 rpm for 20 min. The precipitate was then dispersed into 30 mL of water and ultrasonically treated for 15 min to form an electrophoretic solution. Two pieces of ITO glass were selected as the conductive cathode and anode, respectively. Their ITO conductive surfaces were placed relative to each other, and the distance between the two electrodes was about 3 mm. In the process of electrophoretic deposition, the working voltage of the DC power supply was 10 V. After 30 min of electrophoretic deposition, the uniform distribution of gold nanoparticle thin films on ITO glass occurred. That is, 3D-GNSs were formed.

## 4. Conclusions

In summary, we propose a simple, easy-to-operate, low-cost method that is suitable for the large-scale preparation of 3D-GNSs. Firstly, gold nanoparticles with a yield of almost 100% were prepared by means of multi-step chemical synthesis. Then, a super-hydrophilic 3D-GNS was prepared on ITO glass using the electrophoretic deposition technique. By selecting the appropriate voltage value and deposition time, 3D-GNS thin films with excellent uniformity were obtained. By means of theoretical simulation, the local electric field intensities on the surface of a single gold nanoparticle and multiple nanoparticles were compared. The results show that the coupling effect between nanoparticles can greatly improve the local electric field intensity in the gap region. At the same time, the hot spot effect at the tip of GNSTs provides stronger local electric field enhancement characteristics. The volatilization characteristics of water based on 3D-GNSs were monitored experimentally, and the water volatilization rate of 3D-GNS based on GNSTs was the highest, reaching 8 μL/min. In the SERS signal test, the 3D-GNS SERS substrate based on GNSTs also showed stronger local electric field enhancement characteristics. The minimum detection concentration of R6G molecules on the 3D-GNS SERS substrate based on GNSTs was 10^−12^ M. The 3D-GNSs that we introduced are very simple in terms of chemical synthesis and electrophoretic deposition. At the same time, they have good economic traits and do not rely on complex equipment, endowing them with very competitive industrial prospects.

## Data Availability

Raw data are available from the corresponding author upon request.

## Nomenclature

Abbreviation	Definition
SERS	surface-enhanced Raman scattering
3D-GNS	three-dimensional gold nanostructures
GNSP	gold nanosphere particles
GNST	gold nanostar particles
